# Building capacity for information and communication technology use in global health research and training in China: a qualitative study among Chinese health sciences faculty members

**DOI:** 10.1186/s12961-017-0222-8

**Published:** 2017-06-28

**Authors:** Jie Wang, Abu S. Abdullah, Zhenyu Ma, Hua Fu, Kaiyong Huang, Hongping Yu, Jiaji Wang, Le Cai, Huimin He, Jian Xiao, Lisa Quintiliani, Robert H. Friedman, Li Yang

**Affiliations:** 1 Department of Preventive Medicine & Fudan Health Communication Institute, School of Public Health, Fudan University, Shanghai, 20032 China; 2 Boston University School of Medicine, Boston Medical Center, Boston, MA 02118 United States of America; 3 Duke Global Health Institute, Duke University, Durham, NC 27710 United States of America; 4 Global Health Program, Duke Kunshan University, Jiangsu, 215347 China; 5 School of Public Health, Guangxi Medical University, Nanning, Guangxi 530021 China; 6 School of Public Health, Guilin Medical University, Guilin, Guangxi 541004 China; 7 School of Public Health, Guangzhou Medical University, Guangzhou, Guangdong 511436 China; 8 School of Public Health, Kunming Medical University, Kunming, Yunnan 650500 China; 9 School of Information and Management, Guangxi Medical University, Nanning, Guangxi 530021 China; 10 School of Medicine, Guangxi University of Chinese Medicine, Nanning, Guangxi 530021 China

**Keywords:** Information and communications technology, Training course, Health science faculty members, Global health research

## Abstract

**Background:**

The demand to use information and communications technology (ICT) in education and research has grown fast among researchers and educators working in global health. However, access to ICT resources and the capacity to use them in global health research remains limited among developing country faculty members. In order to address the global health needs and to design an ICT-related training course, we herein explored the Chinese health science faculty members’ perceptions and learning needs for ICT use.

**Methods:**

Nine focus groups discussions (FGDs) were conducted during December 2015 to March 2016, involving 63 faculty members working in areas of health sciences from six universities in China. All FGDs were audio recorded and analysed thematically.

**Results:**

The findings suggest that the understandings of ICT were not clear among many researchers; some thought that the concept of ICT was too wide and ambiguous. Most participants were able to cite examples of ICT application in their research and teaching activities. Positive attitudes and high needs of ICT use and training were common among most participants. Recommendations for ICT training included customised training programmes focusing on a specific specialty, maintaining a balance between theories and practical applications, more emphasis on the application of ICT, and skills in finding the required information from the bulk information available in the internet. Suggestions regarding the format and offering of training included short training programmes, flexible timing, lectures with practicum opportunities, and free of charge or with very minimal cost to the participants. Two participants suggested the linking of ICT-related training courses with faculty members’ year-end assessment and promotion.

**Conclusions:**

This study among health sciences faculty members in China demonstrated a high level of need and interest in learning about ICT use in research and training. The results have important implications for the design and implementation of ICT-related educational programmes in China and other developing countries.

## Background

Information and communication technology (ICT) can be defined as a family of technologies used to process, store and disseminate information, facilitating the performance of information-related human activities, provided by and serving both the public at-large as well as the institutional and business sectors [1]. ICT is increasingly becoming a central component of the learning and teaching environment in most medical schools [2]. Both healthcare and health research delivery have adopted ICT worldwide, especially in research collaboration [3]. ICT has the great potential to increase collaboration in global health research and to build research capacity in developing countries. For example, the use of ICTs can enable space-time flexible global health research collaborations and make it easier to remotely secure pilot/crowd funding for research, find (e-meet) collaborators/mentors, share research databases and jointly publish [4]. ICT directly links health scientists in developing countries with colleagues in the developed countries [5]. Furthermore, research capacity building support for researchers in developing countries has been recognised as both the key and challenging step for long-term global health research [6]. Additionally, ICT has the potential to increase access to training in health research worldwide through online courses and the use of distance learning tools in developing countries [5, 7].

Achieving the above potentials of ICT will depend on an understanding of the current needs, challenges and opportunities of ICT use in developing countries, for the problems and needs are usually different in developing countries than in developed ones [8]. In most developed countries, ICT in health educational and research systems has been imbedded in national strategies, where governments set guidelines that promote the use of ICT [9]. However, in developing countries, access to these ICT resources and the capacity to use them are often lacking in institutions. Additionally, faculty involved in both education and research activities are often required to make major changes in the technology used in their teaching and research systems without sufficient support. Further, although there are examples of ICT use in educational and research systems, such as electronic systems to facilitate literature reviews and massive open online courses that incorporate videos, the use of ICT in health research programmes in developing countries remains quite limited compared to that in developed countries [10–12]. Thus, the potential utility of ICT in health education and research fields is considerable and needs to be fully examined.

There are few studies addressing ICT training and health research or global health research, although some studies related to academic/research activities and ICT training are available. Training of data analysis software, such as SPSS and Nvivo, has been identified as an important demand for ICT training by faculties [7, 13]. Additionally, training of faculty members to keep track of active research projects online, of what ICT tools are currently available to develop research proposals and track progress, and how to use social media to disseminate research have also been identified as important [7]. Several studies showed that organisation support, leadership and effective training and development programmes were the key factors explaining faculty willingness to adopt ICT tools into their teaching and research works [14].

To help faculty adapt to the changes and demands brought by emerging ICTs, some ICT- related training programmes have been developed to train faculty in the use of ICT in the classroom. Online programmes aid teachers in developing their general digital competency for teaching [15] and in optimising the use of tablets in medical education [16]. Nevertheless, future programmes should avoid one-off workshops and hands-on training [17, 18]. Furthermore, training programmes should provide both constant and structured support on the daily issues encountered with ICT [19].

However, studies regarding the use and demand of ICT training in education and global health research in developing countries like China is scarce [12]. Since ICT has great potential in global health research and particularly in helping to build research capability in developing countries, we aimed to design an ICT training course based on the needs and demands of faculty in developing countries. Thus, the findings of this study will have important implications for the design and implementation of ICT training programmes on global health research in developing countries.

In order to design an ICT-related training programme to be used by health sciences faculty members and researchers in China, we herein assessed faculty members’ understanding about ICT and perceptions of their learning needs in relation to use of ICT, what content could be optimally included in an ICT-related training course, what format the course should be offered in, and in what ways such a course can and cannot be used to improve the learning process.

## Methods

### Sample and setting

Since China is one of the world’s largest developing countries (the World Bank website http://www.worldbank.org/en/country/china/overview), and given the rapid development in global health research in Chinese medical universities, an ICT training programme for global health research faculty in China is particularly important and representative. Due to its rapid economic growth and participation in international development work, China weighs heavily in the global health arena. Furthermore, global health capacity is being increasingly developed in medical universities in China. Recently, several new global health centres have been established in universities such as Fudan University, Peking University, Wuhan University and Kunming University. In this endeavour, Chinese collaboration with international universities in global health research has also been steadily growing. Joint programmes in global health research and education in Chinese medical universities have been launched with Duke University, the Graduate Institute of Geneva, Georgetown University and Harvard University, to name a few [20].

Participants were faculty members from selected health sciences universities in China. The majors and research fields of the participants included preclinical medicine, public health, clinical medicine, nursing, pharmacology, general medicine, Chinese traditional medicine, food safety, electronics, and communication engineering. Because of the widening inequalities in economy, education and health between the developed and less developed cities in China, we endeavoured to make the sample representative. Thus, we chose six universities from two cities in developed regions (Guangzhou and Shanghai) and three cities in less developed regions (Guilin, Nanning and Kunming). The settings included Guangxi Medical University (GXMU; Nanning city), Guangxi University of Chinese Medicine (GUCM; Nanning city), Guilin Medical University (GLMU; Guilin city), Guangzhou Medical University (GMU; Guangzhou), Kunming Medical University (KMU; Kunming), and Fudan University (FDU; Shanghai). These universities were conveniently selected due to the established rapport of the principal investigator (PI) with the leadership in these universities.

### Procedures

Focus group discussions (FGDs) were conducted between December 2015 and March 2016. Participants were recruited through research team members serving as liaisons in each participating university. The liaisons were provided with the verbal and written background information about the study and inclusion and exclusion criteria for participants. The liaisons then recruited potential participants to join an upcoming FGD.

### Data collection

Nine FGDs were conducted, involving 63 participants from six universities in five cities of China (Table 1). Four FGDs were conducted in GXMU (each group had 7 participants); in each of the remaining universities, one FGD was conducted. All FGDs were conducted in Mandarin Chinese. A semi-structured FGD guide was developed with reference to the research team’s earlier work [21, 22]. The guide included questions and queries on the following 10 major themes: What is ICT, who should learn ICT, ICT application in their work and field of research, experience with ICT-related educational courses, advantages of ICT training courses, challenges of ICT training courses, suggestions for overcoming the challenges, suggested content and form of optimal ICT training courses, marketing approaches, and cultural factors related to ICT training course design. These themes were developed based on the investigator’s earlier work and the subsequent discussion with the collaborators in Chinese institutions.

**Table 1:** Demographic characteristics of focus group discussion (FGD) participants (*n* = 63)

	GXMU	GUCM	GLMU	GMU	KMU	FDU
	*n* = 28	*n* = 6	*n* = 7	*n* = 7	*n* = 7	*n* = 8
Sex						
Male	16	1	4	4	1	4
Female	12	5	3	3	6	4
Age (mean ± SD)	34.25 ± 4.91	38.33 ± 5.28	36.71 ± 6.40	36.38 ± 10.21	37.67 ± 6.50	38.94 ± 3.21
Major						
Public health	10	1	1	4	6	3
Clinical medicine	5	1	1	1	0	2
Basic science	11	2	1	2	0	3
Others	2	2	4	0	1	0
Degree of Education						
Bachelor	3	0	0	4	4	1
Master	18	4	4	3	2	2
Doctor	7	2	3	0	1	5
Research experience						
5 years or below	1	0	0	1	0	0
Above 5 years	27	6	7	6	7	8

*GXMU* Guangxi Medical University, *GUCM* Guangxi University of Chinese Medicine, *GLMU* Guilin Medical University, *GMU* Guangzhou Medical University, *KMU* Kunming Medical University, *FDU* Fudan University

At each university, two interviewers with previous experience in conducting FGDs acted as moderators. The two interviewers received project-specific information and a focus group guide from the PI and attended an in-person meeting or a 30-minute ‘Skype call’ with the PI to review the guides and to clarify any issues. Two interviewers worked as a team to collect data; one moderated the FGD and the other took detailed notes and recorded the session with a digital voice recorder (after permission had been obtained from the participants). All FGDs were held in a private room within the university and lasted for approximately 120 minutes. The sessions started with the moderators explaining the purpose of the FGD and assuring confidentiality of the data collected for the research project. To compensate for their time, each participant was given a cash amount of RMB 200 ($30). Written informed consent was obtained from each participant. The study was approved by the Ethics Committee of the Guangxi Medical University (No. IRB-SPH-2015: 009).

### Analyses

The interviewers discussed and summarised the content of each FGD and reviewed the notes taken immediately after the interview or discussion [23]. These debriefings were useful to identify the most important themes and to assess the need for any modification in the questions or formats of the subsequent FGDs. The audio recordings were first transcribed in Mandarin Chinese and then translated into English. Two members of the research team identified important information from the transcript according to each interview guide topic and then summarised the key themes. All additional notes taken during the course of the FGDs was examined to identify any additional themes and was incorporated into the analysis. All of the analyses were conducted in English.

## Results

These themes are described below and supplemented by significant statements made by the participants (Table 2).

**Table 2:** Typical statements made by participants by key themes

Questions/Schools	Guangxi University of Chinese Medicine	Guilin Medical University	Guangzhou Medical University	Kunming Medical University	Guangxi Medical University	Fudan University
Understanding and perception of the definition of information and communication technology (ICT)	The medium of information exchange	Technology and life; study and scientific research; technology that brings convenience to people’s lives	Apply ICT to solve practical problems; receive information via terminal server; platform for information exchange; disseminate audio and video material electronically; methods to know the world	A technology of collecting, transmitting, processing and applying information; technology related to programming; transmission of information collected from different sources	The carrier or technology that is related to the transmission of information; technology or information related to electronic computers	All ICT related to computer and high technology; the collecting, processing, output and sharing of information
ICT application in routine research	Literature review; resource sharing among peers; micro-lectures and massive online open courses; remote diagnosis and treatment in medicine; know the newest tendency in one’s research field	Document query; students’ registration system; e-class; catch the trends in one’s research field; two- dimension code which contains extensive information	At present, little technology is used for ICT; some relationship between bioinformatics and ICT; document query; the query of food sources; query and set up of databases; remote consultation; video conferences; teleconferences; examination and assay of cells and blood	Multimedia classes; network examinations, remote operations; conducting experiments with software; measurement and evaluation of the quality and nutritional components of food; electronic survey questionnaires	Relevant websites and public accounts on WeChat to assess trends in relevant research areas; skills on writing papers	Data analysis software in epidemic areas; literature review; attain the trends in research field through public accounts on WeChat and websites; sharing of teaching resources; the platform for communication, discussion and mutual assistance among professional groups; surgical robots
Advantages of ICT-related training courses	The mode is new and original; it is convenient and time saving; there are extensive contents	Learning resources are abundant; contents are original and updated in time; enable participants to learn individually	Extensive contents; convenient and time-saving; learning resources are abundant; can arrange the learning time by oneself and review lessons at any time	One could learn repeatedly; possible to save costs	The communication and convergence between different subjects and different industries can be achieved; learn the trends of research field; practical and pertinent to experimental research; learn what we are interested in	Promote educational equity and share excellent learning resources; enable students to know or learn about their field of interest
Disadvantages of ICT-related training courses	Lack of interactions and practices; some courses’ contents are out of date	Expensive; not updated in time; expensive but of poor quality	Poor quality of courses; lack of interactions; difficult to concentrate on the courses	Lack of interactions; most courses are full of theories but lack practical applications; information not updated regularly	Lack interaction with instructors; insufficient authority and expensive	Lack of interactions; lack of continuities; requires students to be more self-controlled; easy to quit
Suggested content for ICT-related training courses	To stimulate students’ and teachers’ thoughts	Valuable, highly specialised and classified	Include how to collect information and how to use ICT in practical applications	Theoretical framework and basis of ICT; basic computer operation technology, statistical analysis; courses should be designed according to different majors	Novel; timely updated	Basic ICT; design different contents according to different majors and different backgrounds; statistical software and Microsoft Office software
Suggested formats for ICT-related training courses	Should be varied and combine traditional classes with modern teaching methods	Should not rigidly adhere to traditional teaching methods; apply some original teaching methods such as problem-based learning, case analysis	Any format; theories and practical applications should be combined	Basic, foundation and advanced classes could be designed according to students’ learning abilities	Combining lectures with traditional teaching methods; it is not necessary to learn the course material from beginning to the end due to limited time	Basic courses for general audience, videos or lectures; forums and discussion groups could be set up when it comes to the professional or occasional knowledge; applications; resource library; case studies
Suggested duration for ICT-related training courses	Arrange reasonably according to the course contents and different majors	Should be scheduled according to teaching syllabus and course objectives	Should not be accumulated for more than 2 days; 16–20 lessons; one session can last 20–40 minutes according to course contents	20–30 class sessions would be acceptable; each session approximately 30 minutes	Can be decided according to the research needs	Short-period, high efficiency; make use of spare time or weekends
Challenges in ICT-related training courses	Copyright; cost control; how to stimulate researchers’ new ideas and thoughts	Expensive; hard to access; long duration; not regularly updated	Hard to collect effective information quickly; take up too much time; the popularity of the knowledge of ICT; issues on the courses’ fees; process for assessment and evaluation	Clash with faculty member’s teaching commitments; teaching methods are not favoured by faculties; whether one can benefit from course	Copyright; public understanding of ICT; whether practical issues could be solved; time arrangement; whether the course clashes with faculty time arrangements	Time issues: clinicians do not have spare time to attend many courses; the courses should be of short duration but high efficiencient; priorities are always given to free courses
Promotional (marketing) approaches of ICT-related training courses	Seek help from administrative staff or renowned professors; promotion would not be a problem if the course is attractive and original	Link the course to faculty members year-end assessment and career promotion; demo courses can be provided free at first	Emphasise advantages of ICT; link course to faculty promotion; show successful model course; recruit relevant companies to promote the courses; turn to the government for help	Coordinated the course with faculty time-tables; example of achievements, as these speak louder than words; show successful cases to faculty	Show successful cases and course benefits; cooperate with popular relevant companies	Provide course information on the official website of university/school; seek help from administrative staff

### Knowledge of and attitudes towards ICT

When the participants were asked about their perception of the definition of ICT, the process of dealing with information/data was mentioned most often (72 times), which included collecting, processing, exchanging, transmitting, output and sharing of information/data. Some participants related ICT to the Internet (51 times), cell phone (20 times), computers (9 times) and high tech (3 times). Two participants described ICT in general terms, for example: “*technology that brings convenience to people's lives; means and methods to know the world.*”

All participants emphasised that all faculty should learn how to use ICT, especially those who have heavy research tasks or engage in network services and health management. Furthermore, some participants (4/63) mentioned that the whole population should also master some basic elements of ICT, for example: “*Now we are in an age of Internet, ICT is used in almost every field of our society, so everybody needs to learn some basic ICT skills.*”

### Application of ICT in research and education

For application of ICT in research and education, the importance of sharing resources (such as data and keeping records) was mentioned most often (50 times). Application of ICT in research included new research formats (such as data collection, analysis and presentation) (29 times), literature review and document query (22 times), knowing the newest trends in research (15 times), mobile health (12 times) and communication among peers (6 times). ICT applications in the educational field included online courses (in total 93 times, included micro-lectures 35 times and massive online open course 30 times), student registration and management systems (4 times), and the use of computers in multimedia classes (3 times).

Subscribing to a WeChat (the most popular social app in China similar to Facebook) public account and other similar websites was seen as an important approach to keep informed on current issues in their research fields. The use of mobile health for healthcare delivery and management was discussed, including remote diagnosis and treatment in medicine; remote consultation; examination and assay of cells and blood; network examinations; remote operations; the conduct of experiments facilitated by using software; measurement and evaluation of the quality and nutritional components of food; health questionnaires; surgical robots; wearable devices and other real-time information collection and monitoring methods; online medical treatment; WeChat group to manage and educate elderly patients with chronic diseases; and application of medical software to monitor and follow patients’ conditions in real time. The use of ICT in communication among peers included resource sharing, video conferences, teleconferences, relevant international exchange and promotion, discussion and mutual assistance among professional groups.

Some participants from GLMU stated that there were few ICT applications related to their research fields at present. However, other participants provided several examples about the application of ICT in their teaching and research fields.

### Experience of ICT-related training courses

Most participants had received some form of training related to ICT; the content of these courses could be categorised as either education or research. ICT education-related training courses included micro-lectures and massive open online courses (14 times), student registration and management systems (4 times), and making PowerPoint (2 times). ICT research-related training courses included bioinformatics (6 times), document retrieval and database management (8 times), experimental operation (2 times) and statistical training (2 times). It is noteworthy that the faculty members in GMU did not receive much ICT training.

The problems encountered with the ICT-related training courses in which the participants had participated were the lack of interactive as face-to-face communication (11 times); being forced to participate in the ICT-related training courses irrelevant to their major/interest (7 times); the lack of flexibility in the format of the courses (3 times); outdated/impractical content (3 times); not very effective for unmotivated people, but only effective for people who were highly self-motivated (2 times).

To illustrate these themes, one participant said: “*So many times I have been forced to participate in certain courses or trainings that I actually did not feel interested. For example, our school invited someone to give us a lecture on crisis intervention. Basically it was commonplace, with little innovation. The information was conceptual, but not detailed and not updated. So I wonder whether the courses you offer can fix these problems.*”

### Perceived need for ICT-related educational or training courses

The results showed that there was a high perceived need of receiving ICT courses among the participants. Most participants thought it was useful, urgent and important for them to participate in well-designed ICT-related training courses. Only three participants expressed that they did not think it necessary for them to participate in ICT-related training courses. For example, they thought knowing the basic use of software was enough, and therefore they did not need to know the process or principles of how the software works. Further, it was easy to find professional people to help or collaborate with when they really needed to use ICT in teaching and research. This was illustrated as follows (Note: A, B and H used below are the initials of the surnames of the participants):

Interviewee B: “*We really lack that skill at this stage and don't know where to get a better one. Usually what we find could not meet our needs. For example we know some basic elements of ICT, however, when it comes to the elaboration of content in detail we just don't know which is better.*”

Interviewee H: “*Indeed I deem we don't need to learn it. After all, my major is not computer science. We are not able to create and develop a software. All we need to do is to learn how to use it.*”

Interviewee A: “*Yes, I agree with him.*”

Interviewee H: “*Placing an advertisement on a platform contributes to information spreading and thus audiences can find it. It doesn't make sense for us to learn it. It is not the key point.*”

Interviewee A: “*I don't care what the technology itself is, as long as this software is designed to extract key information in relation to my research that would be fine.*”

Interviewee B: “*The result is the only thing that matters. The procedure is not important.*”

### Advantages of ICT-related educational courses

Participants perceived several advantages to participating in ICT-related training courses: flexibility and convenience (14 times), new, original and abundant content (8 times), and time/cost saving (5 times). Participants noted: “*It is possible to choose to learn the attractive part by oneself*”, “*It is possible to arrange the learning time by oneself and review lessons at any time*”, “*Learn the latest development dynamic and the cutting-edge development situation*”. In addition, “*the communication and convergence between different subjects and different industries*” and “*promoting educational equity and sharing marvellous learning resources with each other among colleges and universities*” were also stated as advantages of ICT-related training courses.

### Challenges of ICT-related educational courses

For challenges, the content, form and price of ICT training courses as well as learners’ attitudes were mainly criticised by the participants. The content of the training courses was sometimes out of date, of poor quality and lacked practical applications. For example, several participants mentioned that: “*Some professional courses fail to be updated in time.*” With regards to the course format, there was lack of interaction between the participants and the teachers in ICT training courses. For example: “*We can neither get answers to questions, nor interact with instructors.*” Additionally, some ICT training courses were too expensive. Furthermore, learners found it was too easy to slack off or give up or found it hard to concentrate on the ICT training courses. Participants noted: “*Students involve in the courses with little enthusiasm and it is difficult for students to concentrate on the courses*.”

In addition, most participants believed that, if people doubt the course will be practical, useful, professional or tailored to their teaching and research fields, then this would hinder their participation in ICT training courses (18 times). In addition, being too busy to take the course (9 times), high costs (7 times) and copyright (3 times) were other issues discussed by participants. Participants noted: “*The authority of some online courses is insufficient, and the price of that is too high.*”

Except for the GUCM FGD, all the other five FGDs emphasised that the course duration should kept to a minimum and that there may be scheduling conflicts between the ICT training course and regular teaching activities. Except for KMU and GXMU, the other four FGDs referred to whether the course was free or not, how much it charged and support for the maintenance costs. Finally, copyright issues (i.e. how the copyright of a developed course could be ensured) on the course came up in three FGDs (GUCM, GXMU, and GLMU).

### Overcoming challenges to implement ICT-related educational programmes within the university

For the content of the course, participants thought that the course should be highly specialised and should satisfy different faculty staff needs from different teaching and research fields (18 times). Course content should be based on different staff accessibility, understanding and overall ability in using ICT. Participants thought that the course content should balance theories and practical applications; a short, highly efficient and free ICT training course would always be preferred (16 times). Participants recommended that the timing of the ICT training course should not conflict with other regular teaching and research activities.

### Suggested contents and formats of possible ICT-related educational programmes or training courses

For the contents of ICT training courses, specialised courses according to different majors and different backgrounds (25 times) were emphasised in FGDs at GLMU, KMU and FDU. Furthermore, statistical technology (20 times), teaching people how and where to find the information they need (10 times), and basic computer operation technologies (4 times), were also mentioned as the suggestions about the content of ICT training courses.

“*For example, as a teacher and researcher from clinical medicine, I want to know how to use ICT to find the information I need. You’d better use a concrete instance to let me learn what knowledge or common website will be used, what problem I will encounter, and where to find help.*”

“*Set up different courses according to different majors and different backgrounds.*”

“*Specific teaching contents should be as follows: statistics, the application of statistical software and Microsoft Office such as Word, PowerPoint and so on.*”

For the forms of ICT training courses, combining new teaching methods with traditional teaching methods were emphasised (10 times). Some participants suggested highlighting the novelty and original teaching methods of the forms of the ICT courses.

“*Consider to apply some original teaching methods such as PBL* [problem-based learning]*, cases’ analysis and so on; basic introduction and principles of the courses can be omitted; focus on highlighting the novelty and technology of the courses.*”

“*Public basic courses, videos or lectures; forums and discussion groups could be set up when it comes to the professional or occasional knowledge; associate qualified professionals with third-party organisations to develop an app.*”

### Promotional (marketing) approaches of ICT-related training courses

Showcasing successful cases and benefits of ICT training courses was mentioned the most frequently (8 times), in three FGDs (GLMU, GMU and KMU). Second, using various communication channels as marketing approaches, such as advertisements, forums, information sessions, official websites of university/college/school, and other channels was also mentioned 6 times in the FGDs of GUCM, GMU and FDU. Third, gaining support from the leaders of university/college/school was also considered essential (4 times). Interestingly, participants from GLMU and GMU both advocated linking ICT training courses with faculty members’ year-end assessment and promotion, while participants from FDU were all strongly against and averse to this suggestion.

### Cultural issues in promoting ICT-related training courses

The participants did not frequently mention cultural factors. Only two points appeared often, namely that language translation must be professional and correct to avoid unnecessary misunderstandings and maintaining the balance between theories and practical applications.

In the FGD of GLMU, one participant said: “*Although some colleagues are good in English, so the language problems may be less for them. But if the content of this training course is designed in America, then the language translation problems will appear. You may understand the language when you read the contents, but not necessarily accurate, because the same words can be interpreted differently in different areas.*”

In the FGD of GXMU, participants stated: “*Some Chinese senior aged faculties still stick with the old approach of didactic teaching and only focus to teach theories. But we observe that many educational videos abroad focus on case studies, problem solving, and actual operation. The optimal way to teach is combining theories and practical applications.*”

## Discussion

This study aims to understand the perceptions and needs of health science faculty members who would join ICT-related training courses at six conveniently selected universities from five cities in southern China. We found that most participants thought ICT was quite important to their current academic roles and believed that all faculties should learn ICT and its application for teaching and research. Few participants (n = 4) mentioned that the entire population should also master some basic ICT skills. Although several participants were confused as to the definition of ICT, and considered the concept of ICT as too wide and ambiguous, most participants were able to raise a number of ICT application examples in their work and life.

Participants’ positive attitude and high perceived need towards ICT and ICT-related training courses were common. Participant attitudes were not affected by whether they had had a bad experience of ICT-related training course, how they perceived ICT or their different academic backgrounds. Only three participants at one university questioned the necessity of learning ICT. The recognition and high need for ICT-related training courses may suggest how faculties value ICT and ICT-related training courses in their work, as noted by other studies [24, 25]. This can be interpreted as a positive result in terms of the potential participation in ICT-related training courses in the future [26].

These results have several implications for how an ICT-related educational course might be designed and implemented in China or other developing countries. The course should be practical (connections and applicability in everyday work), which is consistent with findings in other studies [27, 28]. First, recommendations were made for improving the content and format of ICT-related educational courses. The content of the course should be highly specialised to satisfy faculty members’ needs from different teaching and research fields. For example, courses for public health scholars, for clinical medicine or for nursing should have a different focus in content; it should be based on the accessibility, understanding and master ability of ICT of the different staff, which is similar to the findings in other studies [28]. The ICT-related course should contain theories and practical applications. Furthermore, basic computer operation technology, skills training for statistical analyses, and teaching people how and where to find the information they need were also mentioned as suggestions about the content of ICT training courses. Because of the vast volume and complexity of information now available for health science faculties, how to use ICT tools to search, find, evaluate and use such information was also emphasised by American public health faculties [7]. Furthermore, the requirement to be able to conduct statistical analysis through relevant software was also consistent with other ICT training demands studies [7, 13].

The format of the course can combine new teaching methods with traditional ones; short duration, flexible, highly efficient and free ICT training courses will always be preferred, although universities could pay the fees for the faculty members. The ICT training course should not conflict with regular teaching and research activities. Faculties in universities and colleges all have high workloads and limited time; hence, courses of long duration and conflicting with existing teaching and research activities may undermine their effectiveness. Therefore, ICT-related training courses were perceived to be much more effective if faculties that are not overloaded with other working duties and have the necessary capacity to fully participate in the course.

Furthermore, attention needs to be paid in incorporating ICT-related training courses into faculty member career development. Although not universally accepted among our FGD participants, participants at GLMU and GMU advocated for the linking of ICT-related training courses with faculty members’ year-end assessment and promotion, while participants from FDU were all strongly against this link. Thus, whether faculties should be obligated to participate in ICT-related training courses still needs more evidence and clarification. Showing successful cases and benefits of ICT-related training courses as well as the use of various communication channels were recommended as promotional approaches for ICT training programmes.

### Strengths and limitations

The strengths of our study were not only the diverse range of respondents involved in the study in terms of specialty, sex, grade, work experience and location (six different cities in southern China), but also the participation of health research faculties from different medical universities and health sciences schools, allowing the gathering of collectively developed ideas as well as priorities and perspectives from actual experiences.

One limitation of this study was that all data was collected from the health sciences faculty members across five universities in southern China. Therefore, caution is necessary when extrapolating the findings to faculty members of disciplines other than the health sciences and to all universities in China. A further limitation was that all the data were collected by FGDs, and there might be limitations of this method. For example, tendency for certain types of normative opinion to emerge while obscuring some controversial perspectives and certain participants may dominate the whole discussion [29]. However, we referred to purposive sampling, grounded theory, multiple coding, triangulation and respondent validation to confer rigour on qualitative analysis [30]. In addition, the PI had reviewed half of the recorded FGDs in each institution to ensure fidelity in the FGD.

## Conclusions

This study identified several key issues about the use of ICT in global health research and training through a qualitative study among health sciences faculty members in China. It identified the faculty members’ current needs and challenges to use ICT in research and training. This also documented possible contents, formats and promotional (marketing) approaches of ICT-related training courses. These findings should be considered in the design and implementation of an effective ICT-related educational programme in China or other developing countries, so as to achieve the potential of ICT use in global health research and training. Further quantitative studies are needed to gather perspectives from wider audiences across the universities.

## Abbreviations

FDU: Fudan University
FGDs: focus groups discussions
GLMU: Guilin Medical University
GMU: Guangzhou Medical University
GUCM: Guangxi University of Chinese Medicine
GXMU: Guangxi Medical University
ICT: information and communications technology
KMU: Kunming Medical University
PI: principal investigator
